# Lactation Performance and Rumen Fermentation in Dairy Cows Fed a Diet Supplemented with Monensin or Gum Arabic-Nano Montmorillonite Compost

**DOI:** 10.3390/ani14111649

**Published:** 2024-05-31

**Authors:** Salim A. Al Adawi, Hani M. El-Zaiat, Amr S. Morsy, Yosra A. Soltan

**Affiliations:** 1 Department of Animal and Veterinary Sciences, College of Agricultural and Marine Sciences, Sultan Qaboos University, P.O. Box 34, Al-Khod 123, Oman; s124455@student.squ.edu.om; 2 Livestock Research Department, Arid Lands Cultivation Research Institute, City of Scientific Research and Technological Applications, P.O. Box 21934, Alexandria 21934, Egypt; amorsy@srtacity.sci.eg; 3 Department of Animal and Fish Production, Faculty of Agriculture, University of Alexandria, Aflaton St., El-Shatby, P.O. Box 21545, Alexandria 21526, Egypt

**Keywords:** phytochemicals additives, nano-clay, monensin, fermentation, digestibility, health indicators

## Abstract

**Simple Summary:**

Natural feed additives have gained significant scientific attention as growth-promoting substances instead of synthetic antibiotics. The objectives of this study were to develop and investigate the physicochemical properties and effects of Arabic gum–nano montmorillonite (AGNM) compared to monensin as feed additives in Holstein dairy cows. However, the practical application of gum Arabic as a production enhancer for ruminant feeding has not been widely adopted. In this study, the AGNM additive showed great promise for improving rumen fermentation, increasing nutrient digestibility and utilization, and benefiting the environment.

**Abstract:**

The exploration of natural alternatives to antibiotics for enhancing productivity and performance in dairy cows is a crucial objective in farm animal management. This is the first study aimed at developing and evaluating the physicochemical properties and effects of Arabic gum–nano montmorillonite (AGNM) compost compared to ionophore monensin as feed additives on rumen fermentation, blood metabolites, and milk production of Holstein dairy cows. In a replicated 4 × 4 Latin square design, four multiparous mid-lactation Holstein dairy cows with an average body weight of 520 ± 15 kg were enrolled. The dietary treatments included a control diet (basal diet without feed additives), monensin diet [a basal diet supplemented with 35 mg/kg dry matter (DM) monensin], and AGNM diets comprising basal diet supplemented with two levels: low (L-AGNM) at 1.5 g/kg DM, and high (H-AGNM) at 3 g/kg DM. AGNM as a feed additive demonstrated promising physiochemical parameters, including containing highly bioactive components (α-amyrin and lupeol), functional groups (OH and Si-O), and essential mineral contents (Mg^2+^). Supplementations with H-AGNM significantly improved ruminal (*p* = 0.031) concentrations of total volatile fatty acids (VFAs), acetic (*p* = 0.05) and butyric (*p* = 0.05), enhanced (*p* < 0.05) digestibility of fiber and organic matter, while decreased (*p* = 0.013) estimated methane production. However, an increase (*p* = 0.04) in blood high-density lipoprotein levels and decrease (*p* < 0.05) in concentrations of creatinine (CREA), bilirubin (BILT), cholesterol (CHOL), and sodium (Na) were observed with H-AGNM supplementation. Both monensin and H-AGNM improved (*p* = 0.008) feed efficiency compared to L-AGNM; however, neither AGNM nor monensin affected the milk composition or energy status indicators of the dairy cows. The findings of this study highlight the potential of AGNM as a natural candidate to replace monensin in enhancing ruminal VFA production, nutrient digestibility, feed efficiency, blood metabolites, and milk yield in dairy cows.

## 1. Introduction

Ionophores (e.g., monensin), which are a type of carboxylic polyether antibiotic, disrupt the balance of ions across microbial cell membranes, leading to a futile ion cycle within the micro-organisms [1]. Thus, ionophores have been extensively studied as feed additives and proven effective in improving ruminal fermentation and feed efficiency, reducing methane (CH_4_) emissions, and enhancing health and milk production in dairy cattle. However, using ionophores raises concerns regarding antimicrobial resistance and environmental pollution, prompting the search for natural alternatives [1,2], thus the exploration of alternatives to antibiotics for enhancing productivity and performance in cows is a crucial objective in farm animal management nowadays. In ruminant feeding, natural feed additives are an emerging and promising approach resulting in modulating rumen fermentability, enhancing animal health, and promoting productive sustainability [3]. Recently, the European Food Safety Authority [4] authorized gum Arabic as a safe feed additive for the ruminants, for consumers as well as the environment. It is obtained from the dried exudation of the branches and/or stems of natural varieties of *Acacia senegal*. It is designed to be a feed additive, providing functional properties such as emulsification, stabilization, thickening, and gelling in animal feeds [4]. Recently, high-concentrate diets supplemented with gum Arabic had increased total gas production while producing low methane. Additionally, it improved dry matter and neutral detergent fiber digestibility after 24 h of incubation [5].

Gum Arabic has been investigated for its health benefits due to its antibacterial and therapeutic properties, and it can reduce serum cholesterol and triacylglycerol levels and influence the outcomes of various metabolic and non-infectious diseases [6].

Clays such as montmorillonite are widely acknowledged as safe for animal and human consumption also [7]. Montmorillonite is globally accessible and readily available at cost-effective prices. Recently, the organically modified montmorillonite exhibited enhanced physiochemical characteristics and thus a greater affinity for aflatoxin contaminants, positively affecting the ruminal fermentation, pH-buffering agent, reducing ruminal CH_4_ emission, and increasing antimicrobial efficacy against Gram-positive bacteria compared to its natural form [2,8,9]. Recently, numerous innovations in clay-based nano composites have aimed to enhance the physicochemical properties of clays for diverse applications [10]. However, applications of phytochemicals–clay nano composites in the animal production sector remain limited.

This is the first investigation on evaluating gum Arabic–nano montmorillonite compost (AGNM) as a feed additive. Thus, we hypothesized that the potential for combining gum Arabic with nano montmorillonite creates an alternative feed additive capable of enhancing the ruminal fermentation and animal health of dairy cows. Therefore, the objectives of this study are to develop and evaluate the physicochemical properties and potential of AGNM compared to ionophore monensin as feed additives on ruminal fermentation profile, blood attributes, and milk performance of Holstein cows.

## 2. Materials and Methods

The experimental AGNM was developed and characterized at the Nanotechnology and Greenhouse Gases Laboratory, Faculty of Agriculture at Alexandria University, Alexandria, Egypt, while the in vivo experiment was conducted at the Dairy Research Unit located at the Agricultural Experiment Station, Sultan Qaboos University, Muscat, Oman.

### 2.1. Preparation of Gum Arabic-Nano Montmorillonite Compost (AGNM)

An air-dried raw fine powder gum Arabic of certified botanical origin of Sudan Hashab gum Arabic of *Acacia senegalin* was obtained from a local market (Elarayf of Food Industries Company, New Bourg El Arab, Alexandria, Egypt) in airtight zip bags. The natural montmorillonite (Ca-montmorillonite, 95% purity and 741 nm particle size) was purchased (Egypt Bentonite and Derivatives Co., Inc., Alexandria, Egypt). Nano montmorillonite was obtained by grounded natural montmorillonite using a high-energy planetary ball mill (Photon Ball Mill Model PH-BML 912, Photon Scientific Co., Qalyub, Egypt) with a rated rotating speed revolution of 300 turns/min ± 10% and jar rotation of 450 rotations/min ± 10%. The milling lasted for a total of 60 min in a zirconia ball milling jar with a capacity of 100 mL and zirconia balls. To obtain the AGNM compost, the required quantities of the nano montmorillonite and powder gum Arabic (in a ratio of 1:1) were mixed in 20 mL of distilled water and stirred magnetically (at 500 rpm for 12 h at 40 °C) using a stirrer with a hot plate (508-Hotplate-Magnetic, Globe Scientific, Mahwah, NY, USA). The mixture was ultrasonically treated for 60 min using a prop sonication (Model USY2500-1, 2500 W, Bioevopeak, Jinan, China), filtered, washed with distilled water three times, dried in an oven at 50 °C for 48 h.

### 2.2. The Physicochemical Properties of AGNM

The bioactive components of AGNM were identified using gas chromatography/mass spectrometry (GC-MS) (TRACE-1300, Thermo Fisher Scientific Inc., Waltham, MA, USA) with a fused silica DB-5 capillary column (30 m, 0.32 mm id, and 0.25 μm film thickness). The identification of bioactive components was achieved by combining mass spectra and retention index data, utilizing the Main lib library, following the methodology described by Soltan et al. [9]. The AGNM product’s particle size distribution and specific surface area (SSA) were measured using a laser particle analyzer (Better size 2600 particle analyzer; Dandong Better size Scientific Ltd., Dandong, China) equipped with an automatic laser centering function. The particle size distribution was measured at D10, D50, and D90.The elemental composition of the experimental AGNM was analyzed using the energy-dispersive X-ray system (EDX) attached to a scanning electron microscope (SEM; Jeol JSM-6360 LA, 3-1-2 Musashino, Akishima, Tokyo, Japan). Fourier Transform Infra-Red Spectroscopy (FTIR) was used to identify the functional groups present in the experimental clay products. An infrared spectrometer (Shimadzu FTIR-8400S, Osaka, Japan) equipped with a deuterated triglycine sulfate detector and purge gas generator was utilized.

### 2.3. Cows, Experimental Design and Treatments

Four multiparous mid-lactation Holstein dairy cows (520 ± 15 kg body weight; 130 ± 30 DIM; mean ± SD at the onset of the study) were enrolled in a replicated 4 × 4 Latin square design according to the milk yield, dry matter intake (DMI), and body weight (BW) with four 21-day periods and four treatments. Animals were housed individually in free-stall barns, with an open-air shading system with free access to feeders, and fresh water. All cows were fed a basal diet (with a forage-to-concentrate ratio of 40:60) consisting of a mixture of Rhodes grass (*Chloris gayana*) hay and concentrate feed to fulfill the cow’s nutrient requirements according to the NRC [11] guidelines. The basal diet components and chemical analysis are outlined in Table 1. The experimental periods comprised three weeks for treatments, including two weeks for treatment adaptation followed by one week for sampling and data collection.

The dietary treatments consisted of a basal diet without feed additive as the control diet; basal diet supplemented with 35 mg/kg DM monensin sodium (Elanco Animal Health, Greenfield, IN, USA) as a positive control; basal diet supplemented with 1.5 g/kg DM of AGNM (L-AGNM); basal diet supplemented with 3 g/kg DM of AGNM (H-AGNM). Each day, before the morning feeding, the treatment designated for each cow was weighed and subsequently blended into the concentrate mixture, ensuring that every cow received the appropriate amount of their respective treatment. All cows were fed the respective diet twice daily at 9 a.m. and 4 p.m.

### 2.4. Ruminal Fermentation Characteristics Determination

In each collection period and prior to the morning feeding, ruminal samples were collected from each cow using a stomach tube [12]. Rumen samples were filtered through four layers of cheesecloth and promptly assessed for pH using a portable pH meter (HANNA Instruments, Smithfield, VA, USA). For total number of protozoa counting, another 1 mL rumen sample was mixed with 2 mL of methyl green-formalin-saline solution following to the procedure described by Dehority et al. [13]. Another 2 mL subsample was diluted with 1 mL of metaphosphoric acid (25%) and, consequently centrifuged at 15,000× *g* for 20 min at 4 °C for volatile fatty acid (VFA) analysis. The individual VFA concentrations were quantified using gas chromatography (Agilent Technologies instrument, model GC, Agilent 6890 N, Santa Clara, CA, USA) according to El-Zaiat et al. [3]. The net H_2_ produced was estimated following the stoichiometric model conducted according to Wang et al. [14] as Net H_2_ = 2 × (acetate + butyrate + iso-butyrate) − (propionate + iso-valerate + valerate). The CH_4_ (mmol/L) production was calculated using the respective individual VFA according to Moss et al. [15].

### 2.5. Apparent Nutrients Digestibility Determination

During each experimental period, two fecal grab samples (200 g) were obtained 2 h after feeding from each animal at 0900 and 1500 h. At the conclusion of each period, all feeds and fecal samples for each animal were pooled, thoroughly mixed, and stored for future analysis. Nutrients digestibility coefficients were calculated using the lignin content of the feed and fecal samples as an internal marker according to the method mentioned by Van Soest et al. [16]. 

### 2.6. Blood Biochemical Constituents Determination

In each collection period and prior to the morning feeding, a blood sample was drawn from the coccygeal vein for each cow into heparinized tubes before the morning feeding. Blood serum was immediately separated by centrifugation at 2000× *g* for 15 and then frozen (−20 °C) for future processing. The following blood biochemical metabolites were analyzed: total protein (TP), glucose (GLU), alanine aminotransferase (ALT), creatinine (CREA), bilirubin (BILT), blood urea nitrogen(UN), albumin (ALB), triglycerides (TRIGL), cholesterol (CHOL), lactic acid (LACT), lactate dehydrogenase (LDH), gamma-glutamyl Transferase (GGT), aspartate aminotransferase (AST), alkaline phosphatase (ALP), high-density lipoprotein (HDL), creatine kinase (CK), potassium (K), sodium (Na), and chloride (CL). Blood biochemical metabolites were determined using modern COBAS^®^ c111 clinical biochemical analyzer (Roche Diagnostics International Ltd., Roche Rotkreuz, Switzerland). 

### 2.7. Intake, Feed Sampling and Performance

During the collection period, the amount of feed offered, and orts were recorded daily. The amount of feed offered was adjusted weekly based on previous intake to achieve approximately 100 g/kg refusals. Samples of the refused feed were collected daily, and dry matter (DM) was measured to determine daily dry matter intake (DMI).

### 2.8. Milk Production, Sampling and Composition

Cows were milked twice daily at 0500 and 1700 during the experiment. Milk yields were measured at each milking, and samples were collected daily from each cow during the experiment. Milk samples were analyzed immediately for concentrations of protein, lactose, fat, total solid and solid non-fat by Lactoscan Combi milk analyzer (Milkotronic Ltd., Nova Zagora, Bulgaria).

The average daily yield (g/day) of each milk component per milking was determined for individual cows, then summed for the day and averaged across each collection period. Energy-corrected milk yield (ECM) was determined using the method described by Tyrrell and Reid [17] as follows: ECM = (0.327 × kg milk) + (12.95 × kg fat) + (7.65 × kg protein). Additionally, fat corrected milk was calculated using the equation: FCM (kg/day) = 0.4 × milk yield (kg/day) + 15 × fat yield (kg/day). The milk gross energy content (MJ/kg) was calculated according to Tyrrell and Reid [17] as = 184 × 2.204 × [41.63 × fat % + 24.13 × protein % + 21.60 × lactose % − 11.72)/1000]. Milk energy output (MJ/day) was calculated as milk energy content (MJ/kg) × milk yield (kg/day). Feed efficiency was calculated and expressed as milk yield per unit of DMI.

### 2.9. Chemical Analyses

Samples of basal diet, fecal, concentrate mixture and Rhodes grass were dried at 60 °C for 72 h in a forced-air oven and then ground by mill to pass through a 1 mm screen. All ground samples were stored until chemically analyzed for dry matter (DM), ash, ether extract (EE), and crude protein (CP as 6.25 × N%) following the method outlined by AOAC [18]. Neutral detergent fiber (NDF) and acid detergent fiber (ADF) were analyzed following the methods described by Van Soest et al. [16] and Roberston and Van Soest [19], respectively. Non-fiber carbohydrates (NFCs) were calculated using the following equation: NFC (g/kg) = 1000 − (NDF + CP + EE + Ash).

### 2.10. Statistical Analysis

The data from the in vivo experiment underwent statistical analysis following a Latin square design using the MIXED procedure of SAS (SAS Institute Inc., version 9.0, Cary, NC, USA). The model used for analysis is specified as Y_ijkl_ = μ + T_i_ + P_n_ + C_k_ + A_l_ + E_ijkl_, where Y_ijkl_ is the observed response variable, μ is the overall mean, T_i_ is the fixed effect of the treatment, P_n_ is the fixed effect of the period (n = 4), C_k_ is the fixed effect of the carryover effect (if any, to account for potential residual effects from previous treatments), A_l_ is the random effect of the cow, and E_ijkl_ is the random error term associated with each observation. Significance was determined at *p* < 0.05, while trends were acknowledged if *p* < 0.10.

## 3. Results

### 3.1. Bioactive Constituents of AGNM

The gas chromatography/mass spectrometry analysis of the experimental AGNM revealed a diverse array of chemical constituents, as summarized in Table 2. Twelve peaks corresponding to distinct compounds were identified and characterized based on their retention times (RTs), peak areas, molecular formulas, and molecular weights.

Among the compounds detected, α-amyrin (peak 7) exhibited the highest peak area percentage, constituting approximately 56.34% of the total composition. Lupeol (a triterpenoid; peak 11) and oxirane,2,2-dimethyl-3-(3,7,12,16,20-pentamethyl 3,7,11,15,19-heneicosapentaenyl), (peak 4) were also prominent constituents, representing significant proportions of the nano compost of AGNM with peak area percentages of 24.29% and 4.47%, respectively. Several other compounds were present in smaller quantities, including caryophyllene oxide (peak 2), 9,12-octadecadienoic acid (peak 10), and α-D-galactopyranoside (peak 6), each contributing less than 5% to the total composition.

### 3.2. Physicochemical Parameters of Experimental Clay Feed Additives

**Figure 1:**
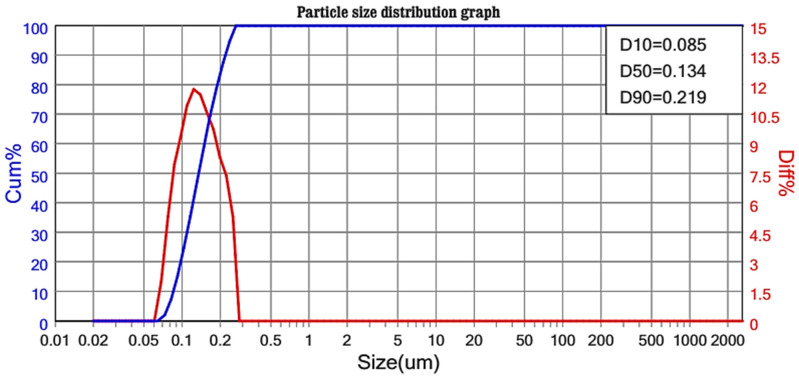
Particle size distribution analysis of the nano compost of gum Arabic extract and montmorillonite (AGNM).

Figure 1 provides a comprehensive analysis of the particle size distribution of AGNM. The analysis indicated a substantial reduction and narrowing in particle size distribution that ranges from 85 to 219 nm, and a value of 146.70 m^2^/g of specific surface area.

Table 3 represents the elemental compositions of GNM determination by EDX. Oxygen and magnesium constitute the predominant elemental components in AGNM, with an atomic percentage concentration of 64.63 ± 0.45, and 14.52 ± 0.16%, respectively. Silicon is a fundamental constituent of montmorillonite and is detected in AGNM at a nuclear percentage concentration of 20.16 ± 0.19%. Aluminum and iron are detected in AGNM, albeit at lower concentrations than oxygen, magnesium, or silicon, with an atomic percentage concentration of 0.55 ± 0.04% and 0.15 ± 0.02%, respectively.

**Figure 2:**
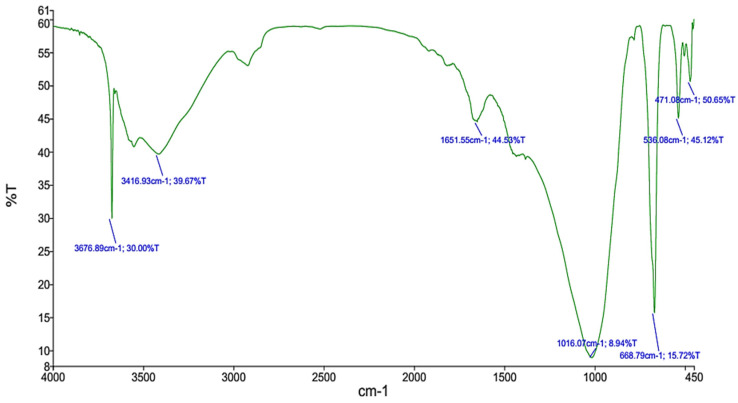
Fourier transform infrared spectroscopy (FTIR) of the nano composite of gum Arabic extract and montmorillonite (AGNM).

Figure 2 shows the functional groups of AGNM detected by FTIR analysis. Different valuable functional groups were detected in the experimental AGNM. Stretching vibrations of hydroxyl (OH) groups were found at high-frequency peaks at 3676 cm^−1^ and 3416 cm^−1^. Si-O and Si-O-Si bonds were detected at medium-frequency bands of 1651 cm^−1^ and 1016 cm^−1^, respectively, and in low-frequency bands of 536 cm^−1^ and 471 cm^−1^. Additionally, a single peak corresponding to bending vibrations of Al-O bonds was observed at 668 cm^−1^.

### 3.3. Effect of AGNM on Ruminal Fermentation Profile

Table 4 presents the supplementation effect of monensin compared to AGNM on ruminal fermentation, protozoal count, and calculated ruminal net H_2_ and CH_4_ emissions of Holstein dairy cows. The concentrations of total VFAs, acetate, and butyrate were the highest values (*p* < 0.05) for cows fed H-AGNM compared to the control, while the highest propionate concentrations (*p* = 0.037) were detected in cows fed monensin compared to cows fed the control diet. No differences were detected among the treated cows in the ruminal concentrations of isobutyric, isovaleric, and valeric.

The monensin supplementation resulted in a significant decrease (*p* = 0.002) in ruminal pH compared to other treatments. Similar reductions (*p* = 0.007) in total protozoal counts for cows fed monensin and H-AGNM compared to those fed on control and L-AGNM. Treatment of H-AGNM resulted in a significant reduction in ruminal net H_2_ (*p* = 0.001) and CH_4_ (*p* = 0.013) emissions compared to the control group, while both monensin and H-AGNM had similar reductions in net H_2_ and CH_4_.

### 3.4. Effect of AGNM on Apparent Nutrients Digestibility

Table 5 shows the supplementation effect of monensin and AGNM on nutrient digestibility of Holstein dairy cows. H-AGNM supplementation led to a significant improvements (*p* < 0.05) in OM and NDF digestibility compared to the control group, while no significant differences were observed in CP, EE, and ADF digestibility among the treatment groups.

### 3.5. Effect of AGNM on Blood Metabolites

The results of Table 6 showed that neither monensin nor AGNM supplementation affected the blood concentrations of TP, ALB, GLU, ALT, AST, UN, TRIGL, CK, LDH, LACT, GGT, K, and CL. Supplementation of H-AGNM resulted in a significant increases (*p* < 0.05) in HDL levels, while decreased (*p* < 0.05) concentrations of CREA, BILT, CHOL and Na compared to the control group.

### 3.6. Effect of AGNM on Feed Intake and Milk Performance

The results in Table 7 reveal the effects of monensin and AGNM supplementations on feed intake, efficiency, milk yield and composition, and energy status indicators in Holstein dairy cows. Notably, cows fed a monensin diet exhibited lower (*p* < 0.0001) DMI compared to the control and L-AGNM groups, while those supplemented with H-AGNM showed intermediate values. Cows supplemented with monensin or H-AGNM demonstrated improved (*p* = 0.001) feed efficiency compared to the L-AGNM group, while H-AGNM enhanced (*p* = 0.022) milk yield compared to L-AGNM treatment. Neither the milk composition nor the energy status indicators were affected by the dietary feed additives.

## 4. Discussion

The gas chromatography/mass spectrometry analysis revealed the presence of highly bioactive components in the experimental AGNM product, such as α-amyrin, lupeol, and oxirane,2,2-dimethyl-3-(3,7,12,16,20-pentamethyl 3,7,11,15,19-heneicosapentaenyl) exhibiting the highest peak areas. These compounds are integral constituents of the diverse phytochemical profile found in plant extracts, including Arabic gum [6]. These findings affirm the successful amalgamation of Arabic gum and montmorillonite in the AGNM formulation. Moreover, the significant presence of these compounds underscores the natural richness of AGNM and its potential health-promoting properties, as α-amyrin and lupeol are triterpenoids known for their diverse biological activities, including anti-inflammatory, antioxidant, antimicrobial, anticancer, and immune-modulatory properties [9,20]. The presence of these phytochemicals in AGNM not only validates the manufacturing process but also hints at the expected promising biological effects of AGNM as a feed additive for dairy animals.

No data were available from the literature regarding the presence of small particles or nanoparticles in gum Arabic, as the examination of five batches of gum Arabic indicated that about 15% of its particles exhibited a diameter of around 63 μm, as determined through sieve analysis [4]. Our previous study, utilizing the same source of raw montmorillonite before any modifications, recorded an average particle size of 741.6 nm [2]. Therefore, the observed smaller nanoparticle size range in AGNM, ranging from 85 to 219 nm, suggests effective modification and nano structuring processes. This reduction and narrowing in particle size distribution are indicative of effective modification processes, likely resulting from the incorporation of gum Arabic extract and the nano structuring of montmorillonite. Such transformations are crucial as they enhanced the AGNM specific surface area and consequently reactivity, which can significantly impact its properties and applications [21].

The EDX analysis findings align with those obtained from FTIR results, underscoring the consistency across analytical methods. The heightened oxygen levels detected by EDX analysis in AGNM confirm the presence of oxygen-rich functional groups detected by FTIR, such as OH, Si-O, and O-Si-O bonds found at high and medium frequency bands [22]. Additionally, the detection of Si-O bonds at peaks of 1651 cm^−1^ and 1016 cm^−1^ and O-Si-O at the 471 cm^−1^ peak further supports the presence of montmorillonite within AGNM, as silicon is a fundamental constituent of montmorillonite [23]. The presence of Al-O bonds at 668 cm^−1^ was likely corresponded to the aluminum content inherent in montmorillonite. Despite its lower abundance, aluminum can still wield considerable influence over the properties of AGNM, especially concerning its surface chemistry and reactivity. Gum Arabic is known to contain a higher content of magnesium than montmorillonite [6]. The presence of magnesium at high concentration in AGNM suggested the incorporation of magnesium ions into the montmorillonite lattice during the modification process [2]. Generally, the convergence between EDX and FTIR analyses may confirm the successful integration of montmorillonite into the nano composite structure of AGNM, validating its structural integrity and composition.

The nutritional effects of AGNM were evaluated compared to monensin in dairy cows, concerning the ruminal fermentation parameters. It appears both AGNM and monensin affect the VFA profile differently. AGNM appears to exert a selective influence on ruminal microbial growth, promoting certain micro-organisms while inhibiting others. The substantial increases in total VFA, acetate, and butyrate concentrations observed in the H-AGNM group compared to both the monensin and control groups support this notion. This could potentially be attributed to the antimicrobial and antioxidant properties of phytochemical components present in AGNM, such as α-amyrin and lupeol [6]. The observed enhancements in total VFAs in cows fed the H-AGNM group may primarily be due to increased acetate production, as acetate typically represents the predominant proportion of VFAs generated through ruminal fermentation [1].

The increase in ruminal pH caused by H-AGNM can create favorable conditions for acetate and fiber-degrading micro-organisms [1]. This also may explain the enhancement in the total tract digestibility of OM and NDF of cows fed H-AGNM compared to other treatments. The presence of interlayer –OH functional groups may facilitate the capture of CO_2_ generated during ruminal fermentation, leading to the formation of –HCO_3_ [24]. This process, alongside the buffering capacity of montmorillonite, in turn, could contribute to an increase in ruminal pH.

The monensin is known to reduce the ruminal pH. This may create an environment conducive to the proliferation of acid-tolerant microbial populations, which may explain the increases in propionate concentrations caused by a monensin diet [1].

It is worth noting that most ruminal acetate and butyrate producers are sensitive to acidic conditions, while acid-tolerant species tend to favor propionate production [25]. This could potentially explain the observed enhancements in propionate concentrations resulting from monensin treatment. The traditional mechanism of action attributed to monensin involves the inhibition of CH_4_ emissions, primarily through the reduction of protozoal populations [26]. However, our study revealed notable distinctions between the effects of H-AGNM and monensin treatments on CH_4_ and H_2_ inhibition, despite their similarities in overall inhibition levels. This suggests that while both treatments may achieve comparable reductions in CH_4_ and H_2_ emissions, they likely operate via distinct modes of action, as evidenced by their differential impacts on the ruminal VFA profile.

The supplementation effects of monensin and AGNM on blood metabolites in Holstein dairy cows were assessed through various parameters. Notably, several significant differences were observed, indicating potential impacts on metabolic processes and health status. In addition, many blood biochemical constituents remained in the normal healthy range for dairy animals. The maintenance of numerous blood biochemical constituents within the normal healthy range, including TP, ALB, GLU, ALT, AST, UN, TRIGL, CK, LDH, LACT, GGT, K, and CL, reflects the overall health and metabolic stability of the dairy animals in this study [27]. These findings suggest that neither the supplementation with H-AGNM nor monensin induced any significant metabolic disturbances or adverse effects on the blood biochemistry of the animals. Maintaining these parameters within normal limits is crucial for ensuring the well-being and physiological homeostasis of dairy animals, indicating the safety and tolerability of the dietary interventions.

Supplementation with H-AGNM led to notable alterations in certain blood biochemical parameters compared to other treatments. Specifically, there was a significant increase in HDL levels, indicating a potentially favorable effect on lipid metabolism [27]. Conversely, the reduction in CREA levels suggests potential improvements in kidney function or muscle metabolism, while the decrease in BILT concentrations may indicate enhanced liver health. The lowered CHOL levels could signify improvements in lipid metabolism and cardiovascular health. Additionally, the decrease in Na levels may reflect alterations in electrolyte balance or fluid regulation [27].

Unexpectedly, neither H-AGNM nor monensin affected blood GLU concentrations, despite improvements in the VFA profile compared to other diets. One possible explanation for this discrepancy could be the improvement in GLU utilization to enhance milk yield by H-AGNM. It is known that GLU is a crucial substrate for synthesizing lactose, a major component of milk, and increased milk production may lead to higher utilization of GLU by the mammary gland for lactose synthesis [9]. The enhancement in the feed efficiency of cows fed an H-AGNM diet partly confirms the positive energy supply and GLU utilization.

While the relatively small number of experimental animals was a limitation of our study, it still provides valuable insights into the effects of AGNM on dairy cow performance and health. Notably, cows receiving the monensin diet exhibited significantly lower DMI compared to the control, indicating a slow rate of ruminal degradation of dietary fiber [28] and consequently CH_4_ emission. Clays, such as zeolite, show promise in enhancing the health of transition dairy cows, offering a new avenue for dairy farms to prevent postpartum pathologies. However, they did not have an impact on milk yield [29]. In the current study, the observed significant increase in milk yield among cows supplemented with H-AGNM compared to those receiving L-AGNM treatment underscores the importance of selecting the optimal dosage to maximize the positive effects on milk production in dairy cows. This finding suggests that the dosage of AGNM supplementation plays a critical role in modulating milk yield, with higher doses potentially exerting a more pronounced impact on dairy cow productivity. Neither the composition of milk nor the energy status indicators were significantly affected by the dietary feed additives. This suggested that while monensin and H-AGNM may enhance feed efficiency and milk yield, they do not exert discernible effects on the overall nutritional composition of milk or the energy balance of the animals.

## 5. Conclusions

The utilization of AGNM as a feed additive demonstrated promising physiochemical parameters, including highly bioactive components such as α-amyrin and lupeol, functional groups such as OH and Si-O, and essential mineral contents such as Mg. These characteristics led to significant improvements in ruminal fermentation profile, CH_4_ inhibition, nutrient digestibility, blood metabolites, and milk yield in dairy cows supplemented with AGNM at a high level (4 g/kg DM). Notably, AGNM supplementation enhanced VFA production and improved the feed efficiency of dairy cows. Furthermore, AGNM supplementation resulted in favorable alterations in blood metabolites. However, neither AGNM nor monensin affected the milk composition or energy status indicators of dairy cows. These findings underscore the potential of AGNM as a viable natural alternative to monensin as a feed additive in dairy cow nutrition, offering promising avenues for further research and application to improve herd health and productivity.

## Figures and Tables

**Table 1:** Experimental feed ingredients and chemical compositions (g/kg dry matter).

Item	Concentrate Feed Mixture	Rhodes Grass Hay	Experimental Basal Diet ^1^
Ingredients			
Rhodes grass hay	-	-	400
Ground yellow corn	541	-	324
Soybean meal	230	-	138
Wheat bran	206	-	124
Limestone	14.0	-	8.00
Sodium chloride	6.00	-	4.00
Mineral mixture ^2^	3.00	-	2.00
Chemical composition			
Ash	115	91.9	111
Organic matter	885	908	902
Crude protein	173	92.4	151
Extract ether	32.7	17.8	25.8
Neutral detergent fiber	213	604	368
Acid detergent fiber	107	486	245
Non-fiber carbohydrates ^3^	445	193	343

^1^ Consisted of 400 g Rhodes grass hay/kg DM and 600 g concentrate feed mixture /kg DM; ^2^ Mixture provided the following: Ca, 1.00%; P, 0.60%; Se, 0.3 mg/kg; vit A, 8800 IU/kg; vit D, 2200 IU/kg and vit E, 33 IU/kg. ^3^ Non-fiber carbohydrates (g/kg) = 1000 − (ash + crude protein +extract ether + neutral detergent fiber).

**Table 2:** Chemical constituents identified by gas chromatography/mass spectrometry analysis of the nano compost of gum Arabic extract and montmorillonite (AGNM).

Peaks	Compounds	RT (min) ^a^	Peak Area (%)	Molecular Formula	Molecular Weight
1	(3S,3aR,3bR,4S,7R,7aR)-4-Isopropyl-3,7-dimethyloctahydro-1H-cyclopenta[1,3]cyclopropa[1,2]benzen-3-ol	19.47	0.83	C_15_H_26_O	222
2	Caryophyllene oxide	21.11	0.83	C_15_H_24_O	220
3	9-(3,3-Dimethyloxiran-2-yl)-2,7-dimethylnona-2,6-dien-1-ol	35.09	3.92	C_15_H_26_O_2_	238
4	Oxirane,2,2-dimethyl-3-(3,7,12,16,20-pentamethyl 3,7,11,15,19-heneicosapentaenyl)-, (all-E)	35.23	4.47	C_30_H_50_O	426
5	2H-Pyran,2-(7-heptadecynyloxy)tetrahydro-	35.80	0.82	C_22_H_40_O_2_	336
6	à-D-galactopyranoside,methyl2,3-BIS-O-(trimethylsilyl)-,cyclic butylboronate	49.85	2.74	C_17_H_37_BO_6_Si_2_	404
7	à-Amyrin	50.55	56.34	C_30_H_50_O	426
8	t-Butyl-(2-[3-(2,2-dimethyl-6-methylene-cyclohexyl)-propyl]-[1,3]dithian- 2-yl)-dimethyl-silane	53.56	0.91	C_22_H_42_S_2_Si	398
9	4H-1-Benzopyran-4-one,2-(3,4-dimethoxyphenyl)-3,5 dihydroxy -7- methoxy	53.70	1.10	C_18_H_16_O_7_	344
10	9,12-octadecadienoic acid(z,z)-,2,3bis[(trimethylsilyl)oxy] propyl ester	54.05	2.67	C_27_H_54_O_4_Si_2_	498
11	Lupeol	54.25	24.29	C_30_H_50_O	426
12	7,10,13-Eicosatrienoic acid, methyl ester	55.54	1.10	C_21_H_36_O_2_	320

^a^ RT = retention time.

**Table 3:** The elemental compositions of the nano composite of gum Arabic extract and montmorillonite (AGNM) using energy dispersive X-ray spectrum (EDX).

Element (Atomic %) *	AGNM
O^−2^	64.63 ± 0.45
Mg^+2^	14.52 ± 0.16
Al^+3^	0.55 ± 0.04
Si^+4^	20.16 ± 0.19
Fe^+2^	0.15 ± 0.02

* The values are given as atomic percentage concentrations (atomic %) and have been normalized to 100%.

**Table 4:** Supplementation effect of monensin or nano composite of gum Arabic extract and montmorillonite (AGNM) on ruminal fermentation profile of Holstein dairy cows.

	Treatments					
	Control	Monensin	AGNM		SEM	*p* Value
Variables			L-AGNM	H-AGNM		
VFA (mmol/L)						
Total	80.1 ^c^	90.0 ^ab^	81.6 ^ab^	98.5 ^a^	8.55	0.032
Acetic	52.9 ^c^	58.1 ^b^	52.8 ^c^	65.2 ^a^	5.24	0.054
Propionic	13.6 ^b^	17.2 ^a^	14.7 ^ab^	15.7 ^ab^	0.86	0.037
Butyric	10.7 ^c^	11.0 ^bc^	10.9 ^c^	14.4 ^a^	0.84	0.055
Isobutyric	0.74	0.87	0.72	0.88	0.01	0.348
Valeric	1.01	1.32	1.12	1.40	0.03	0.179
Isovaleric	1.14	1.55	1.21	1.37	0.03	0.373
Ruminal pH	6.22 ^a^	6.04 ^b^	6.14 ^a^	6.20 ^a^	0.01	0.002
Protozoa × 10^5^	3.46 ^a^	2.56 ^b^	3.12 ^a^	2.65 ^b^	0.24	0.007
Estimated Net H_2_	113 ^a^	84.8 ^ab^	111 ^a^	68.6 ^b^	9.58	0.009
Estimated CH_4_, mmol/L	24.4 ^a^	18.1 ^ab^	24.1 ^a^	16.3 ^b^	3.42	0.013

L-AGNM = AGNM supplemented at 1.5 g/kg DM, H-AGNM = AGNM supplemented at 3 g/kg DM, SEM = standard error of the mean, VFAs = Volatile fatty acids. Within a row, means with different letters (a, b and c) are significantly different (*p* < 0.05).

**Table 5:** Supplementation effect of monensin or nano composite of gum Arabic extract and montmorillonite (AGNM) on apparent nutrients digestibility of Holstein dairy cows.

	Treatments					
	Control	Monensin	AGNM		SEM	*p* Value
Variables			L-AGNM	H-AGNM		
Organic matter, %	61.4 ^b^	66.2 ^b^	64.5 ^b^	74.7 ^a^	3.18	0.005
Crud protein, %	62.2	65.9	65.7	66.0	3.03	0.322
Ether extract, %	56.5	57.7	57.4	58.2	3.52	0.924
Neutral detergent fiber, %	56.4 ^b^	55.1 ^b^	60.1 ^b^	73.3 ^a^	2.88	0.0004
Acid detergent fiber, %	52.0	48.5	50.0	52.3	4.28	0.587

L-AGNM = AGNM supplemented at 1.5 g/kg DM, H-AGNM = AGNM supplemented at 3 g/kg DM, SEM = standard error of the mean, Within a row, means with different letters (a and b) are significantly different (*p* < 0.05).

**Table 6:** Supplementation effect of monensin or nano composite of gum Arabic extract and montmorillonit (AGNM) on blood metabolites of Holstein dairy cows.

	Treatments					
	Control	Monensin	AGNM		SEM	*p* Value
Variables			L-AGNM	H-AGNM		
Total protein g/L	103	99.9	96.6	97.8	8.08	0.670
Albumin g/L	43.9	42.6	43.6	43.5	1.57	0.749
Glucose g/L	59.4	57.2	53.0	54.9	2.22	0.687
Alanine Transaminase U/L	23.2	22.1	23.1	24.1	2.11	0.646
Aspartate Transferase U/L	101	95.9	99.3	103	7.78	0.861
Creatinine umol/L	74.8 ^a^	71.6 ^ab^	74.7 ^a^	60.3 ^b^	5.33	0.024
Bilirubin umol/L	1.20 ^a^	1.12 ^a^	0.95 ^b^	0.30 ^c^	0.004	0.022
Blood urea nitrogen mmol/L	4.54	4.21	4.07	4.25	0.34	0.352
Triglycerides mmol/L	0.08	0.07	0.08	0.06	0.01	0.111
Cholesterol mmol/L	3.64 ^a^	3.38 ^a^	3.57 ^a^	2.59 ^b^	0.21	0.001
High density lipoprotein mmol/L	4.15 ^ab^	3.85 ^b^	3.78 ^b^	4.98 ^a^	0.49	0.046
Creatine kinase U/L	194	172	189	186	14.2	0.621
Blood lactate mmol/L	1.29	1.25	1.32	1.39	0.12	0.493
Lactate dehydrogenase U/L	1012	887	1050	928	90.4	0.133
Gamma-glutamyl transferase U/L	33.7	32.5	28.0	26.0	5.39	0.239
Potassium mmol/L	4.62	4.50	4.66	4.76	0.30	0.700
Sodium mmol/L	104 ^a^	105 ^a^	102 ^a^	88.9 ^b^	6.79	0.041
Chloride mmol/L	97.1	93.4	94.7	94.2	4.34	0.673

L-AGNM = AGNM supplemented at 1.5 g/kg DM, H-AGNM = AGNM supplemented at 3 g/kg DM, SEM = standard error of the mean, Within a row, means with different letters (a, b and c) are significantly different (*p* < 0.05).

**Table 7:** Supplementation effect of monensin or nano composite of gum Arabic extract and montmorillonite (AGNM) on feed intake, and milk performance and composition of Holstein dairy cows.

	Treatments					
Variables	Control	Monensin	AGNM		SEM	*p* Value
			L-AGNM	H-AGNM		
Dry matter intake kg/day	17.6 ^a^	16.9 ^c^	17.4 ^ab^	17.2 ^bc^	0.05	<0.001
Milk yield kg/day	24.3 ^ab^	24.1 ^ab^	23.5 ^b^	24.5 ^a^	0.35	0.022
Feed efficiency	1.38 ^ab^	1.41 ^a^	1.35 ^b^	1.42 ^a^	0.004	0.001
Milk composition (%)						
Fat	3.18	3.21	3.15	2.86	0.08	0.611
Protein	3.14	3.12	3.14	3.11	0.02	0.996
lactose	4.47	4.68	4.46	4.42	0.02	0.321
Solid not fat	8.63	8.53	8.60	8.53	0.15	0.990
Total solids	10.5	10.8	10.8	10.9	0.37	0.950
Energy status indicators						
ECM	23.9	22.4	22.4	22.3	0.95	0.399
FCM	21.9	20.6	20.6	20.3	0.98	0.426
MEC (MJ/kg)	2.73	2.79	2.72	2.59	0.02	0.629
MEO (MJ/day)	68.4	65.4	64.1	63.3	3.96	0.569

L-AGNM = AGNM supplemented at 1.5 g/kg DM, H-AGNM = AGNM supplemented at 3 g/kg DM, SEM = standard error of the mean, ECM = energy corrected milk, FCM = fat corrected milk, MEC = milk energy content and MEO = milk energy output. Within a row, means with different letters (a, b and c) are significantly different (*p* < 0.05).

## Data Availability

Data are contained within the article.

## References

[1] Benchaar C., Greathead H. (2011). Essential oils and opportunities to mitigate enteric methane emissions from ruminants. Anim. Feed Sci. Technol..

[2] Soltan Y., Morsy A., Hashem N., Elazab M., Sultan M., Marey H., Lail G.A.E., El-Desoky N., Hosny N., Mahdy A. (2021). Modified nano-Montmorillonite and monensin modulate in vitro ruminal fermentation, nutrient degradability, and methanogenesis differently. Animals.

[3] El-Zaiat H.M., Elshafie I. (2022). Elshafie; Waleed Al-Marzooqi. Effects of Neem (*Azadirachta indica*) Leaf Powder Supplementation on Rumen Fermentation, Feed Intake, Apparent Digestibility and Performance in Omani Sheep. Animals.

[4] Bampidis V., Azimonti G., Bastos M.D.L., Christensen H., Dusemund B., Fašmon Durjava M., Kouba M., López-Alonso M., López Puente S., EFSA Panel on Additives and Products or Substances used in Animal Feed (FEEDAP) (2022). Safety and efficacy of a feed additive consisting of acacia gum (gum Arabic) for all animal species (A.I.P.G. Association for International Promotion of Gums). EFSA J..

[5] Han H., Yamanaka S., Tsukahara T., Hotta Y., Takagi T., Kumagai H. (2021). In vitro ruminal fermentation characteristics of gum arabic under concentrate and forage substrate conditions. Anim. Sci. J..

[6] Patel S., Goyal A. (2015). Applications of Natural Polymer Gum Arabic: A Review. Int. J. Food Prop..

[7] Maki C.R., Haney S., Wang M., Ward S.H., Bailey R.H. (2017). Calcium montmorillonite clay for the reduction of aflatoxin residues in milk and dairy products. Dairy Vet. Sci. J..

[8] Ullah N., Ali Z., Ullah S., Khan A.S., Adalat B., Nasrullah A., Alsaadi M., Ahmad Z. (2022). Synthesis of activated carbon-surfactant modified montmorillonite clay-alginate composite membrane for methylene blue adsorption. Chemosphere.

[9] Soltan Y.A., Morsy A.S., Hashem N.M., Sallam S.M. (2021). Boswellia sacra resin as a phytogenic feed supplement to enhance ruminal fermentation, milk yield, and metabolic energy status of early lactating goats. Anim. Feed Sci. Technol..

[10] Umejuru E.C., Mashifana T., Kandjou V., Amani-Beni M., Sadeghifar H., Fayazi M., Karimi-Maleh H., Sithole T. (2023). Application of zeolite based nanocomposites for wastewater remediation: Evaluating newer and environmentally benign approaches. Environ. Res..

[11] NRC (2001). Nutrient Requirements of Dairy Cattle.

[12] Ramos-Morales E., Arco-Pérez A., Martín-García A.I., Yáñez-Ruiz D.R., Frutos P., Hervás G. (2014). Use of stomach tubing as an alternative to rumen cannulation to study ruminal fermentation and microbiota in sheep and goats. Anim. Feed Sci. Technol..

[13] Dehority B., Damron W., McLaren J. (1983). Occurrence of the rumen ciliate *Oligoisotricha bubali* in domestic cattle (Bostaurus). Appl. Environ. Microb..

[14] Wang M., Sun X.Z., Janssen P.H., Tang S.X., Tan Z.L. (2014). Responses of methane production and fermentation pathways to the increased dissolved hydrogen concentration generated by eight substrates in in vitro ruminal cultures. Anim. Feed Sci. Technol..

[15] Moss A.R., Jouany J.-P., Newbold J. (2000). Methane production by ruminants: Its contribution to global warming. Ann. Zootech..

[16] Van Soest P.J., Robertson J.B., Lewis B.A. (1991). Methods for dietary fiber, neutral detergent fiber, and non-starch polysaccharides in relation to animal nutrition. J. Dairy Sci..

[17] Tyrrell H.F., Reid J.T. (1965). Prediction of the energy value of cows milk. J. Dairy Sci..

[18] AOAC (2000). Official Methods of Analysis.

[19] Roberston J.B., Van Soest P.J., James W.P.T., Theander O. (1981). Chapter 9: The detergent system of analysis. The Analysis of Dietary Fiber in Food.

[20] Roy N.K., Parama D., Banik K., Bordoloi D., Devi A.K., Thakur K.K., Padmavathi G., Shakibaei M., Fan L., Sethi G. (2019). An update on pharmacological potential of boswellic acids against chronic diseases. Int. J. Mol. Sci..

[21] El-Nile A., Elazab M., El-Zaiat H., El-Azrak K.E.D., Elkomy A., Sallam S., Soltan Y. (2021). In vitro and in vivo assessment of dietary supplementation of both natural or nano-zeolite in goat diets: Effects on ruminal fermentation and nutrients digestibility. Animals.

[22] Al Kausor M., Sen Gupta S., Bhattacharyya K.G., Chakrabortty D. (2022). Montmorillonite and modified montmorillonite as adsorbents for removal of water soluble organic dyes: A review on current status of the art. Inorg. Chem. Commun..

[23] Magaña S.M., Quintana P., Aguilar D.H., Toledo J.A., Ángeles-Chávez C., Cortés M.A., León L., Freile-Pelegrín Y., López T., Torres Sánchez R.M. (2008). Antibacterial activity of montmorillonites modified with silver. J. Mol. Catal. A Chem..

[24] Chouikhi N., Cecilia J.A., Vilarrasa-Garcia E., Besghaier S., Chlendi M., Franco Duro F.I., Rodriguez Castellon E., Bagane M. (2019). CO_2_ adsorption of materials synthesized from clay minerals: A review. Minerals.

[25] Spanghero M., Zanfi C., Fabbro E., Scicutella N., Camellini C. (2008). Effect of a blend of essential oils on some end products of in vitro rumen fermentation. Anim. Feed Sci. Technol..

[26] Patra A., Park T., Kim M., Yu Z. (2017). Rumen methanogens and mitigation of methane emission by anti-methanogenic compounds and substances. J. Anim. Sci. Biotechnol..

[27] Melendez P., Severino K., Marin M.P., Duchens M. (2018). The effect of a product with three gluconeogenic precursors during the transition period on blood metabolites and milk yield in Chilean Holstein cattle. J. Appl. Anim. Res..

[28] Soltan Y., Abdalla Filho A., Abdalla A., Berenchtein B., Schiavinatto P., Costa C. (2021). Replacing maize with low tannin sorghum grains: Lamb growth performance, microbial protein synthesis and enteric methane production. Anim. Prod. Sci..

[29] Giurgiu O.V., Berean D.I., Ionescu A., Ciupe M.S., Cimpean C.R., Radu C.I., Bitica D.G., Bogdan S., Bogdan M.L. (2023). The effect of oral administration of zeolite on the energy metabolism and reproductive health of Romanian spotted breed in advanced gestation and post-partum period. Vet. Anim. Sci..

